# Infant mortality by color or race from Rondônia, Brazilian Amazon

**DOI:** 10.1590/S1518-8787.2017051006411

**Published:** 2017-03-29

**Authors:** Caroline Gava, Andrey Moreira Cardoso, Paulo Cesar Basta

**Affiliations:** I Programa de Pós-Graduação de Epidemiologia em Saúde Pública. Escola Nacional de Saúde Pública Sérgio Arouca. Fundação Oswaldo Cruz. Rio de Janeiro, RJ, Brasil; II Departamento de Endemias Samuel Pessoa. Escola Nacional de Saúde Pública Sérgio Arouca. Fundação Oswaldo Cruz. Rio de Janeiro, RJ, Brasil

**Keywords:** Infant Mortality, Birth Certificates, Death Certificates, Ethnicity and Health, Health Inequalities, Vital Statistics

## Abstract

**OBJECTIVE:**

To analyze the quality of records for live births and infant deaths and to estimate the infant mortality rate for skin color or race, in order to explore possible racial inequalities in health.

**METHODS:**

Descriptive study that analyzed the quality of records of the Live Births Information System and Mortality Information System in Rondônia, Brazilian Amazonian, between 2006-2009. The infant mortality rates were estimated for skin color or race with the direct method and corrected by: (1) proportional distribution of deaths with missing data related to skin color or race; and (2) application of correction factors. We also calculated proportional mortality by causes and age groups.

**RESULTS:**

The capture of live births and deaths improved in relation to 2006-2007, which required lower correction factors to estimate infant mortality rate. The risk of death of indigenous infant (31.3/1,000 live births) was higher than that noted for the other skin color or race groups, exceeding by 60% the infant mortality rate in Rondônia (19.9/1,000 live births). Black children had the highest neonatal infant mortality rate, while the indigenous had the highest post-neonatal infant mortality rate. Among the indigenous deaths, 15.2% were due to ill-defined causes, while the other groups did not exceed 5.4%. The proportional infant mortality due to infectious and parasitic diseases was higher among indigenous children (12.1%), while among black children it occurred due to external causes (8.7%).

**CONCLUSIONS:**

Expressive inequalities in infant mortality were noted between skin color or race categories, more unfavorable for indigenous infants. Correction factors proposed in the literature lack to consider differences in underreporting of deaths for skin color or race. The specific correction among the color or race categories would likely result in exacerbation of the observed inequalities.

## RESUMO

**OBJETIVO:**

Analisar a qualidade dos registros de nascidos vivos e de óbitos infantis e estimar a taxa de mortalidade infantil segundo cor ou raça, a fim de explorar iniquidades étnico-raciais em saúde.

**MÉTODOS:**

Estudo descritivo que analisou a qualidade dos registros do Sistema de Informações sobre Nascidos Vivos e do Sistema de Informações sobre Mortalidade em Rondônia, Amazônia brasileira, entre 2006-2009. As taxas de mortalidade infantil foram estimadas nas categorias de cor ou raça, pelo método direto, e corrigidas por: (1) distribuição proporcional dos óbitos com cor ou raça ignorada; e (2) aplicação de fatores de correção. Efetuou-se também o cálculo da mortalidade proporcional por causas e grupos etários.

**RESULTADOS:**

Entre 2008-2009, a captação de nascimentos e óbitos melhorou em relação aos anos de 2006-2007, requerendo fatores de correção menores para estimar a taxa de mortalidade infantil. O risco de morte de crianças indígenas (31,3/1.000 nascidos vivos) foi maior que o registrado nos demais grupos de cor ou raça, excedendo em 60% a mortalidade infantil média no estado (19,9/1.000 nascidos vivos). As crianças pretas apresentaram as maiores taxas de mortalidade infantil neonatal, enquanto as indígenas apresentaram as maiores taxas de mortalidade infantil pós-neonatal. Observou-se que 15,2% dos óbitos indígenas foram por causas mal definidas, enquanto nos demais grupos não ultrapassaram 5,4%. A mortalidade infantil proporcional por doenças infecciosas e parasitárias foi maior entre indígenas, ao passo que entre crianças pretas, sobressaíram as causas externas (8,7%).

**CONCLUSÕES:**

Observaram-se expressivas iniquidades na mortalidade infantil entre as categorias de cor ou raça, com situação mais desfavorável às crianças indígenas. Os fatores de correção propostos na literatura não consideram diferenças na subenumeração de óbitos entre as categorias de cor ou raça. A correção específica entre as categorias de cor ou raça provavelmente resultaria em exacerbação das iniquidades observadas.

## INTRODUCTION

The infant mortality rate (IMR) is a synthetic indicator that reflects the life conditions of the population, the quality of birth assistance and newborn, and the most common health problems in the first year of life^18^ ^,^ ^a^. According to Victora et al.^18^, infant mortality decreased in the last three decades in Brazil, mainly due to the increase in the socioeconomic and sanitary conditions and access to health services. Nevertheless, recent studies have shown that indigenous infant mortality remains high, above other color or race groups, exceeding at least twice the average in the country^5^ ^,^ ^6^ ^,^ ^8^ ^,^ ^10^ ^,^ ^b^.

Reliable estimates of infant mortality from direct method calculations depend essentially on the quality of live birth and death records in a given population. To use the vital data contained in the Health Information Systems, we also need to consider coverage of events, non-duplication of records, completeness of variables and data measurement^12^.

Thus, recent investigations indicate a continuous influence of underreporting of infant deaths and live births on IMR, which impairs the validity of the produced estimates and the elucidation of factors associated with the risk of death in the first year of life^2^ ^,^ ^9^ ^,^ ^c^, particularly in regions and population groups in social disadvantage^5^ ^,^ ^12^. To correct such deficiencies, some authors have developed alternative strategies, which include prior and critical analyzes about the quality of vital databases and indirect estimates, as well as the application of correction factors before calculating infant mortality^5^ ^,^ ^9^ ^,^ ^c^.

Despite the increase of ethnic-racial scientific publications with skin color or race variable in Health Information Systems^7^ ^,^ ^11^ ^,^ ^15^ ^,^ ^17^, few studies have analyzed the indigenous infant mortality^5^ ^,^ ^6^ ^,^ ^8^ ^,^ ^12^ ^,^ ^14^ ^,^ ^b^. In addition, none of the identified studies used underreporting of deaths and live births. We believe that this analysis can deepen the knowledge about the health situation of indigenous infants in Brazil and broaden the discussion about color or race inequities.

This study aimed to analyze the quality of records of live births and deaths in Rondônia and estimate the infant mortality rate according to color or race, in order to explore possible ethnic-racial inequalities in health.

## METHODS

Rondônia is located in the northern region of Brazil and divided into 52 municipalities. According to data from the last Demographic Census (2010)^d^, its population has 1,562,409 inhabitants, of which 13,076 are indigenous, less than 1% of the total. Although less expressive, the indigenous population of Rondônia shows great ethnic diversity with more than 50 ethnic groups^e^.

We carried out a descriptive epidemiological study of infant mortality, according to color or race in the state of Rondônia. For such, the databases of the Live Births Information System (SINASC) and the Mortality Information System (SIM) were available by the Department of Informatics of the National Health System (DATASUS) for the period of January 1, 2006 to December 12, 2009. However, the data referring to 2005 and 2010 were used to calculate average indicators for triennials to correct underreporting of infant deaths.

As of 2011, the color or race variable has changed in SINASC. According to SINASC’s 2011 technical document^f^, the mother’s color or race started being collected rather than that of the child in the Living Birth Declaration. For this reason, and to ensure data comparability, we did not analyze data from the most recent years. In this study, the Asian color or race category was excluded from the analyzes due to the small number of events recorded in SIM (n = 2; 0.1%) and SINASC (n = 178; 0.2%).

We estimated IMR by the direct method according to color or race categories, considering the ratio numerator and denominator, respectively, the number of deaths and live births reported and classified in each color or race category, multiplied by 1,000. In order to incorporate to the IMR all live births and deaths in infants under one year into the information systems, we distributed live births and deaths with color or race ignored proportionally to the color or race. Thus, we obtained infant mortality rates by proportionally modified color or race (IMRprop)^5^.

To obtain a more accurate underreporting of infant deaths and live births in Brazil, we followed Frias et al.^9^ recommendations. These authors stratified the general coefficient of standardized mortality (GMCpad) into categories (≥ 1 and < 2; ≥ 2 and < 3; ≥ 3 and < 4; ≥ 4 and < 5; ≥ 5 and < 5.5; ≥ 5.5 per 1,000 inhabitants). The authors established a correction factor per Federative Unit (FU) for each category, separating deaths in children under and over one year of age.

In our study, in the GMCpad calculation, we used the Brazilian population as reference. The population data used in the denominators originated from IBGE, based on censuses and intersensorial estimates, available on DATASUS^g^. In order to obtain GMCpad estimates for 2006, 2007, 2008 and 2009 in Rondônia, the average of this coefficient was used for three years, grouping the following years respectively: 2005 to 2007; 2006 to 2008; 2007 to 2009; and 2008 to 2010. The correction factor for deaths under one year old was then applied, according to the categories proposed by Frias et al.^9^

The authors, in order to correct the information on live births, have established strata for the Live Birth Ratio (LBR) reported and estimated (< 0.5; ≥ 0.5 and < 0.6; ≥ 0.6 and < 0.7; ≥ 0.7 and <0.8; ≥ 0.8 and < 0.9; ≥ 0.9), and also established a correction factor for each category per FU^9^. In our study, the LBR provided by the Indicators and Basic Data of Brazil (IDB)^g^ and the correction factor corresponding per year were used.

We calculated the corrected IMR by color or race category. The ratios between the estimated rates for each color or race category were also calculated with reference to the mean IMR and the estimates for the mixed children, who had lower IMR.

Similarly, we calculated IMR by color or race, considering the early neonatal (death up to six days of life), late neonatal (deaths from seven to 27 days of life) and post-neonatal (deaths from 28 to 364 days of life).

Finally, Chapters of the Tenth Revision of the International Statistical Classification of Diseases and Related Health Problems (ICD10) described proportional infant mortality, according to color or race categories. We structured the data in spreadsheets and analyzed them in the program Statistical Package for the Social Sciences version 16.0.

The Research Ethics Committee of the National School of Public Health approved this study, protocol n. 191/09, CAAE: 0203.0.031.000.09.

## RESULTS

From 2006 to 2009, SINASC recorded 100,617 live births, with an average of 25,015 births per year. The proportion of live births with ignored color or race was 1.5%, 1.1%, 2.3% and 3.2%, in 2006, 2007, 2008 and 2009, respectively. In SIM, 1,813 deaths of children under one year were reported, corresponding to an annual average of 454 infant deaths. The proportion of deaths with ignored color or race was 9.4%, 18.0%, 19.7% and 13.6% in 2006, 2007, 2008 and 2009, respectively.

The data quality analysis in SINASC and SIM, through LBR and GMCpad, showed that in 2008 and 2009 an improvement of live births and deaths occurred in relation to 2006 and 2007. Table 1 shows the parameters related to the quality of vital databases and the correction factors.

**Table 1:** Number of live births (LB) recorded in the SINASC, deaths of children under one year registered in the SIM, LB ratio (LBR), correction factor (CF) of LB, standardized general mortality coefficient (GMCpad), CF of deaths in children under 1 year, corrected LB and corrected deaths of less than 1 year. Rondônia, Brazil, 2006-2009.

Year	LB^a^	Deaths under 1 year^b^	LBR	CF LB	GMCpad	CF Deaths under 1 year	Corrected LB	Deaths corrected for under 1 year
2006	24,875	480	0.8	1.17	5.4	1.31	29,104	629
2007	22,960	445	0.8	1.17	4.7	1.55	26,863	690
2008	26,754	436	0.9	1.06	5.6	1.02	28,359	445
2009	26,028	452	0.9	1.06	6.0	1.02	27,590	461

Total	100,617	1,813	-	-	-	-	111,916	2,224

SINASC: Live Birth Information System; SIM: Mortality Information System

^a^ We excluded a total of 178 Asian color or race records.

^b^ We excluded two deaths of Asian color or race records.

Table 2 shows the IMR related to color or race and their variants. IMR increased in all skin color or race categories when we applied the proportional distribution of deaths and live births with ignored color or race and correction factors. The mean IMR among indigenous and black children was significantly higher than the mean IMR of the state and the mean IMR among the mixed children (Table 3). We observed that a significant change in the pattern of infant mortality among all color or race categories occurred in 2007, especially among black and indigenous children, with a reversal in the number of deaths (Table 2).

**Table 2:** Number of Live Births (LB) and infant deaths with color or race in SIM and SINASC and corrected and IMR estimated by direct method (crude) and corrected by proportional distribution of LB and deaths with undeclared color or race (IMRprop) or additionally by application of the correction factor IMRCF, in relation to color or race and year of occurrence. Rondônia, Brazil, 2006-2009.

Year	Skin color or race	No correction			Proportional correction			Proportional correction + CF		
		
		LB (n)	Deaths (n)	IMR_crude_	LB (n)	Deaths (n)	IMR_prop_	LB (n)	Deaths (n)	IMR_CF_
2006	White	9,807	200	20.4	9,952	221	22.2	11,644	289	24.8
	Mixed	14,184	222	15.7	14,394	245	17.0	16,841	321	19.1
	Black	294	5	17.0	298	6	18.5	349	7	20.7
	Indigenous	227	8	35.2	230	9	38.3	269	12	42.9
	Total	24,875	480	19.3	24,875	480	19.3	29,104	629	21.6
2007	White	8,515	173	20.3	8,612	211	24.5	10,076	326	32.4
	Mixed	13,630	180	13.2	13,786	220	15.9	16,130	341	21.1
	Black	241	8	33.2	244	10	39.6	285	15	52.5
	Indigenous	315	4	12.7	319	5	15.1	373	7	20.1
	Total	22,960	445	19.4	22,960	445	19.4	26,863	690	25.7
2008	White	9,760	176	18.0	9,991	219	21.9	10,590	224	21.1
	Mixed	15,741	158	10.0	16,114	197	12.2	17,081	201	11.8
	Black	260	6	23.1	266	7	27.7	282	8	26.7
	Indigenous	374	10	26.7	383	12	32.3	406	13	31.1
	Total	26,754	436	16.3	26,754	436	16.3	28,359	445	15.7
2009	White	9,340	175	18.7	9,684	202	21.0	10,266	206	20.2
	Mixed	15,208	201	13.2	15,708	233	14.8	16,650	237	14.2
	Black	270	4	14.8	279	5	16.5	296	5	15.9
	Indigenous	381	11	28.9	394	13	32.2	418	13	31.0
	Total	26,028	452	17.4	26,028	452	17.4	27,590	461	16.7

SINASC: Live Birth Information System; SIM: Mortality Information System

**Table 3:** Quadrennial infant mortality rates (IMR) (2006-2009) crude and corrected proportionally and by application of the correction factor and rate ratios (in relation to the State and the mixed category, of less value), by color or race. Rondônia, Brazil, 2006-2009.

	White	Black	Mixed	Indigenous	Rondônia
IMR_crude_	19.4	22.0	13.0	25.9	18.1
IMR_prop_	22.4	25.6	15.0	29.5	18.1
IMR_CF_	24.6	28.9	16.5	31.3	19.9

Rate ratio (Ref.: IMR Rondônia)^a^	1.2	1.5	0.8	1.6	-

Rate ratio (Ref.: mixed category)^b^	1.5	1.7	1.0	1.9	-

^a^ Rate ratio between the quadrennial IMR of each color or race category in relation to the IMR of the State of Rondônia (reference).

^b^ Rate ratio between the quadrennial IMR of each of the other color or race categories in relation to the IMR of the mixed category (reference), which showed the lowest values.

Approximately 1/3 of infant deaths occurred in the post-neonatal. However, among the indigenous infants, a higher proportion of deaths occurred in the post-neonatal (46.3%) (Table 4). Analyzing the IMR during the neonatal, the black color or race category had the highest number, exceeding the corresponding rate in mixed children by 40%. IMR in the post-neonatal period between indigenous and black children exceeded 3.0 and 2.4 times the corresponding rate among mixed children, respectively (non-tabulated data).

**Table 4:** Number of deaths, corrected infant mortality rates (IMR) and proportional mortality by age group, by color or race. Rondônia, Brazil, 2006-2009.

Period	Category	0-6 days			7-27 days			28-364 days		
		
		n	IMR	% in total of deaths	n	IMR	% in total of deaths	n	IMR	% in total of deaths
2006	White	158	13.5	54.5	45	3.8	15.5	87	7.4	30.0
	Mixed	197	11.7	61.2	51	3.0	15.8	74	4.4	23.0
	Black	3	8.3	42.9	1	4.1	14.3	3	8.3	42.9
	Indigenous	3	10.8	27.3	1	5.4	9.1	7	26.8	63.6
	Total	360	12.4	57.3	98	3.4	15.6	170	5.9	27.1
2007	White	161	15.9	49.2	38	3.7	11.6	128	12.7	39.1
	Mixed	168	10.4	49.3	62	3.8	18.2	111	6.9	32.6
	Black	11	39.3	73.3	0	-	0.0	4	13.2	26.7
	Indigenous	6	15.0	75.0	0	-	0.0	2	5.0	25.0
	Total	346	12.9	50.1	99	3.7	14.3	245	9.1	35.5
2008	White	90	8.5	40.2	41	3.9	18.3	93	8.8	41.5
	Mixed	127	7.4	63.2	27	1.6	13.4	47	2.8	23.4
	Black	4	13.5	50.0	0	-	0.0	4	13.2	50.0
	Indigenous	2	5.0	22.2	1	3.3	11.1	6	15.3	66.7
	Total	225	7.9	50.7	69	2.4	15.5	150	5.3	33.8
2009	White	93	9.1	45.1	32	3.2	15.5	81	7.9	39.3
	Mixed	127	7.6	53.6	48	2.9	20.3	62	3.7	26.2
	Black	2	8.0	50.0	0	-	0.0	2	7.9	50.0
	Indigenous	8	19.7	61.5	1	2.9	7.7	4	8.4	30.8
	Total	231	8.4	50.0	82	3	17.7	149	5.4	32.3
2006-2009	White	502	11.8	47.9	156	3.7	14.9	389	9.1	37.2
	Mixed	619	9.3	56.2	188	2.8	17.1	294	4.4	26.7
	Black	20	16.5	58.8	1	0.8	2.9	13	10.7	38.2
	Indigenous	19	13	46.3	3	2	7.3	19	13.0	46.3
	Total	1,162	10.4	52.2	348	3.1	15.6	714	6.4	32.1

Proportional mortality due to causes was not similarly distributed among the ICD10 chapters in color or race categories (Table 5). In general, there was a predominance of perinatal conditions in all color or race categories, and the percentage of black and indigenous children was 47.8% and 45.5%, respectively. It is noteworthy that 15.2% of indigenous infant deaths were due to ill-defined causes, while in other color or race categories the proportion of deaths due to ill-defined causes did not exceed 5.4% (white). Among indigenous children, higher proportional mortality due to infectious and parasitic diseases also occurred (12.1%) compared to white (5.8%), black (4.3%) and mixed children (7.2%). It is also noteworthy the higher proportion of deaths due to external causes in black children, 8.7%, while in other color or race categories, mortality due to external causes did not exceed 3.0%. Among black children, there was higher proportion of deaths due to respiratory system diseases, approximately three times higher than the average of other color or race categories.

**Table 5:** Basic causes of death in children under one year, according to color or race categories and ICD10 chapters. Rondônia, Brazil, 2006-2009.

ICD-10 chapter	White		Black		Mixed		Indigenous		Ignored		Total	
					
	n	%	n	%	n	%	n	%	n	%	n	%
I. Infectious and parasitic diseases	42	5.8	1	4.3	55	7.2	4	12.1	19	7.0	121	6.7
II. Neoplasia (tumors)	2	0.3	0	0	3	0.4	0	0	0	0	5	0.3
III. Hematologic diseases	2	0.3	0	0	2	0.3	0	0	0	0	4	0.2
IV. Nutritional and metabolic endocrine diseases	19	2.6	0	0	14	1.8	1	3.0	4	1.5	38	2.1
VI. Diseases of the nervous system	19	2.6	1	4.3	5	0.7	0	0	3	1.1	28	1.5
IX. Diseases of the circulatory system	10	1.4	1	4.3	12	1.6	0	0	2	0.7	25	1.4
X. Diseases of the respiratory system	48	6.6	4	17.4	35	4.6	1	3.0	16	5.9	104	5.7
XI. Diseases of the digestive system	6	0.8	0	0	9	1.2	1	3.0	2	0.7	18	1.0
XIV. Diseases of the genitourinary system	4	0.6	0	0	1	0.1	0	0	0	0	5	0.3
XVI. Some perinatal conditions	397	54.8	11	47.8	442	57.9	15	45.5	158	57.9	1,023	56.3
XVII. Congenital malformation	120	16.6	2	8.7	133	17.4	5	15.2	59	21.6	319	17.6
XVIII. Unclear	39	5.4	1	4.3	34	4.5	5	15.2	7	2.6	86	4.7
XX. External causes	16	2.2	2	8.7	18	2.4	1	3.0	3	1.1	40	2.2

Total	724	100	23	100	763	100	33	100	273	100	1,816	100

Source: SIM – DATASUS.

## DISCUSSION

The corrected infant mortality rate in the state of Rondônia from 2006 to 2009 (19.9/1,000 LB) was similar to the national IMR average for the same period (18.2/1,000 LB)^g^.

Taken together, our analyzes suggest limitations in SIM and SINASC databases of Rondônia for the studied period. The low coverage or underreporting of deaths and live births, the problems in the completeness of some variables, and the low quality of information on the basic death causes, especially in 2006 and 2007, are highlighted. The results indicate that these limitations differently affected the color or race categories. According to some authors^4^ ^,^ ^16^ differences recorded in infant mortality indicators among different population groups may indicate that strategies for preventing infant deaths lack to being applied equally.

In spite of the limitations, we noticed that in 2008 and 2009 the capture of vital events improved, since the GMC standardized by age and LBR presented values closer to the expected standards, resulting in the application of minor correction factors. Nevertheless, we observed significant inequalities in infant mortality among the color or race categories, with the worst situation recorded among black and indigenous children.

Despite the effort made by Szwarcwald et al.^c^ and Frias et al.^9^, when elaborating correction factors for vital statistics in the North and Northeast regions, we disregarded differences in the underreporting of deaths among color or race categories. Therefore, even after applying the correction factors in this study, our estimates lack to correct probable inequalities in the underreporting of deaths and LB among the color or race categories. Applying specific correction factors by color or race category would probably result in an exacerbation of observed inequities. However, these factors are not yet available to assess the real dimension of child mortality inequalities in Brazil.

Unlike the other categories of color or race, from 2008 to 2009 the IMR among black children reduced almost 70%. This phenomenon occurred in only one category that traditionally suffers discrimination and social disadvantages; therefore, an effective improvement in the care of black children is unlikely. On the contrary, this reduction may be indicative of problems related to the underreporting of infant deaths, or merely to a random variation in the number of deaths among black children in 2009.

In general, approximately 2/3 of deaths of children under one year occurred in Rondônia in the neonatal period. According to Victora et al.^16^, deaths were almost entirely due to perinatal causes and congenital anomalies (also called endogenous causes). Therefore, they related to problems in pregnancy and childbirth, or are due to maternal genetic disorders. The first and second cause of deaths of children under one year was perinatal conditions and congenital malformations, respectively. Together, these causes accounted for almost 3/4 of the proportional mortality by ICD10 chapters.

In turn, mortality in the post-neonatal period presumably relates to environmental factors, infectious diseases, malnutrition, among other causes, also known as exogenous causes^16^. In general, deaths corresponded to approximately 1/3 of all infant deaths, in Rondônia. However, the highest proportions of deaths in the post-neonatal were recorded between indigenous and black children, indicating inequalities in health services and suggesting precarious socioeconomic, environmental and health conditions.

Reinforcing the hypothesis of inequalities in health services, the highest proportion of deaths due to ill-defined causes was among indigenous children (15.2%), approximately three times higher than observed in other color or race categories. Deaths due to ill-defined causes indicate poor quality or lack of medical care in any context. When it comes to ill-defined deaths in children under one year, the situation is even more critical. The Ministry of Health^h^ recommends that epidemiological surveillance and monitoring of infant and maternal mortality be carried out throughout the national territory, aiming to investigate deaths due to ill-defined causes, since they impair the analysis of factors that influence mortality and, consequently, makes it difficult to elaborate intervention actions^i^.

According to Victora et al.^18^, survey of maternal and child health in Brazil, infant mortality rates have declined substantially in the last three decades, with a decrease of 5.5% per year between the 1980s and 1990s, and 4.4% per year since 2000, until 20 deaths per 1,000 LB in 2008. This trend of sustained and continuous reduction is due to improved socioeconomic conditions, as well as increased access to health services and improved management of respiratory infections and diarrhea in children^3^ ^,^ ^j^. Therefore, perinatal conditions and congenital malformations have become the leading causes of death in infants nationwide, especially in the most economically developed regions.

Interestingly, Rondônia, a state in the North, where the health situation is historically unfavorable when compared to the great urban centers, showed a pattern of infant mortality with predominance of deaths due to perinatal conditions and congenital malformations. This finding reinforces our hypothesis that there was a underreporting of deaths and live births in the information systems, during the studied period. Underreporting probably focused on the poorest populations with the least access to health services, possibly in more remote areas.

Given that more than 90% of the live births occurred in a hospital environment^1^, the probability of deaths in the neonatal period could have been higher than in the post-neonatal, when the children were already in their communities of origin. This issue would potentially be more relevant in indigenous populations living in isolated rural areas. Thus, the occurrence of a post-neonatal death in these communities would be more likely to underreporting due to the more remote contact with the health services. In some situations, the burials of the children would occur locally, without the knowledge of the authorities. This fact may have underestimated the IMR for these populations.

The prevalence of infant deaths as a result of perinatal conditions and congenital malformations in the national setting poses major challenges to the Unified Health System, especially regarding the improvement of prenatal care, delivery and puerperium programs^1^ ^,^ ^13^, especially in the poorest regions and segments of the population.

Finally, to increase the knowledge about the issues related to underreporting of infant deaths, we suggest to incorporate the ethnicity perspective in the studies on infant mortality; or to differentiate correction factors by types of household (urban or rural), which are known to influence access to health services. Therefore, more subsidies improve maternal and childcare programs, with the consequent expansion of health services access, especially for ethnic minorities, guaranteeing equity and universal service to the specific needs of each segment of the population.

## Footnotes

a Barata RB. Condições de saúde da população brasileira. In: Giovanella L, Escorel S, Lobato LVC, Noronha JC, Carvalho AI, organizadores. Política e sistema de saúde no Brasil. Rio de Janeiro: Editora FIOCRUZ; 2008. p.167-213.

b Basta PC, Orellana JDY, Arantes R. Perfil epidemiológico dos povos indígenas no Brasil: notas sobre agravos selecionados. In: Garnelo L; Pontes AL, organizadores. Saúde indígena: uma introdução ao tema. Brasília (DF): MEC,SECADI; 2012. p.60-106.

c Szwarcwald CL, Morais Neto OL, Frias PG, Souza Junior PRB, Cortez Escalante JJ, Lima RB, et al. Busca ativa de óbitos e nascimentos no Nordeste e na Amazônia Legal: estimação da mortalidade infantil nos municípios brasileiros. In: Ministério da Saúde, Secretaria de Vigilância em Saúde, Departamento de Análise de Situação em Saúde. Saúde Brasil 2010: uma análise da situação de saúde e de evidências selecionadas de impacto de ações de vigilância em saúde. Brasília (DF): Ministério da Saúde; 2011. p.99-114.

d Instituto Brasileiro de Geografia e Estatística. Censo 2010: resultados. Rio de Janeiro: IBGE; 2012 [cited 2015 May 8]. Available from: http://censo2010.ibge.gov.br/resultados

e Ministério da Saúde (BR), Secretaria Especial de Saúde Indígena. Sistema de Informação da Atenção à Saúde Indígena SIASI: dados populacionais indígenas por diversos parâmetros de análise Rondônia. Brasília (DF); 2013 [cited 2015 May 8]. Available from: http://portalsaude.saude.gov.br/index.php/o-ministerio/principal/secretarias/secretaria-sesai/mais-sobre-sesai/9518-destaques

f Ministério da Saúde (BR), Secretaria de Vigilância em Saúde, Coordenação Geral de Informações e Análises Epidemiológicas. Consolidação do Sistema de Informações Sobre Nascidos Vivos – 2011. Brasília (DF); 2013 [cited 2016 Nov 29]. Available from: http://tabnet.datasus.gov.br/cgi/sinasc/Consolida_Sinasc_2011.pdf

g Ministério da Saúde (BR), Departamento de Informática do SUS – DATASUS. Informações de Saúde (TABNET). [cited 2015 Mar 3]. Available from: http://www2.datasus.gov.br/DATASUS/index.php?area=02

h Ministério da Saúde (BR). Portaria GM nº 1.172, de 15 de junho de 2004. Regulamenta a NOB SUS 01/96 no que se refere às competências da União, Estados, Municípios e Distrito Federal, na área de Vigilância em Saúde, define a sistemática de financiamento e dá outras providências. Brasília (DF); 2004 [cited 2016 Nov 29]. Available from: http://www.funasa.gov.br/site/wp-content/files_mf/Port_1172_2004.pdf

i Ministério da Saúde, Secretaria de Vigilância em Saúde, Secretaria de Atenção à Saúde. Manual de vigilância do óbito infantil e fetal e do Comitê de Prevenção do Óbito Infantil e Fetal. 2. ed. Brasília (DF); 2009.

j Ministério da Saúde, Secretaria de Vigilância em Saúde, Departamento de Análise de Situação de Saúde. Saúde Brasil 2009: uma análise da situação de saúde e da agenda nacional e internacional de prioridades em saúde. Brasília (DF); 2010. (Série G. Estatística e Informação em Saúde).
